# miR-210 promotes the anti-inflammatory phenotype and M2 polarization in murine macrophages

**DOI:** 10.3389/fimmu.2025.1633163

**Published:** 2025-08-05

**Authors:** Carmen Alexandra Neculachi, Evelyn-Gabriela Nastase-Rusu, Laudy Cherry, Catalina Iolanda Marinescu-Colan, Spyros Tastsoglou, Bogdan Paul Cosman, Alina Madalina Popa, Cristina Panciuc, Germana Zaccagnini, Sergiu Bogdan Catrina, Maya Simionescu, Fabio Martelli, Mihai Bogdan Preda, Alexandrina Burlacu

**Affiliations:** ^1^ Department of Stem Cell Biology, Institute of Cellular Biology and Pathology “Nicolae Simionescu” Bucharest, Bucharest, Romania; ^2^ Molecular Cardiology Laboratory, Istituto di Ricovero e Cura a Carattere Scientifico (IRCCS) Policlinico San Donato, Milan, Italy; ^3^ Department of Molecular Medicine and Surgery, Karolinska Institute, Stockholm, Sweden; ^4^ Center for Diabetes, Academic Specialist Centrum, Stockholm, Sweden

**Keywords:** miR-210, macrophages, polarization, inflammation, metabolism, cell cycle

## Abstract

**Introduction:**

Macrophages play fundamental roles in immune regulation and tissue homeostasis, serving as one of the primary cell types that orchestrate tissue repair after injury. MiR-210 is a hypoxia-inducible, small non-coding RNA involved in regulating metabolic adaptation and inflammatory responses during normal repair processes. However, its role in macrophage polarization is not fully understood. Here, we report the impact of miR-210 deletion on macrophage polarization towards a pro-reparatory phenotype.

**Methods:**

Bone marrow-derived macrophages were obtained from miR-210 knockout (KO) and wild-type (WT) mice and polarized toward the pro-reparative M2 phenotype. The transcriptomic profile of these cells, as well as their phagocytic capacity, cell energy phenotype, and cytokine production were assessed to determine the impact of miR-210 on the macrophage polarization process into a M2-like phenotype.

**Results:**

Compared with their WT counterparts, miR-210 KO M0 macrophages presented a reduced glycolytic activity and a diminished metabolic flexibility. However, miR-210 KO cells exhibited increased phagocytosis in both M0 and M2 states, potentially as an adaptive response to their metabolic limitations. Transcriptomic analysis revealed distinct clustering between the M0 and M2 states, along with several notable differences in the transcriptional patterns between the two genotypes. Analysis of differentially expressed genes indicated an increased pro-inflammatory state in resting miR-210 KO macrophages compared to WT control cells. These data were further confirmed by the higher levels of IL-6, TNF-α, and IL-1b secreted by miR-210 KO M0 macrophages compared to WT cells. Analysis of the biological processes activated during the polarization process towards the M2 phenotype revealed an incomplete polarization of miR-210 KO cells, which may be attributed, at least in part, to reduced activation of mitotic regulators, leading to slower cell cycle progression and diminished proliferation.

**Discussion:**

Our data offers new insights into the role of miR-210 in promoting a macrophage shift toward the anti-inflammatory, pro-reparative M2 phenotype. The fine-tuned involvement of miR-210 in immune responses may have potential implications for chronic inflammation, immune dysfunction, and tissue repair.

## 1 Introduction

Macrophages are critical components of the innate immune system, being the main responding cells to injury and infections (1). Microenvironmental signals influence macrophage behavior, driving their differentiation into distinct phenotypes: M1-like macrophages, with pro-inflammatory properties, and M2-like macrophages, with anti-inflammatory functions. This polarization is a critical factor in the progression of various diseases, including chronic inflammatory disorders (2–4), sepsis (5), autoimmune diseases (2, 3, 6), and metabolic syndrome (7, 8).

As essential post-transcriptional regulators of gene expression, non-coding RNAs influence numerous cellular processes, including inflammatory responses (9). In turn, inflammatory lesions are commonly linked to tissue hypoxia, making hypoxia and inflammation closely interconnected in a variety of human diseases. Among hypoxia-responsive non-coding RNAs, miR-210 is one of the best characterized. It plays important roles in diverse cellular processes related to inflammation, being an important player in the crosstalk between hypoxia and inflammation (10). MiR-210 regulates genes associated with glycolysis and oxidative phosphorylation, which are both essential for macrophage activation and polarization (11–15).

The metabolic profile of macrophages is generally linked to their functional status. M1-like macrophages preferentially utilize glycolysis to promptly generate energy and ROS, thus supporting their pro-inflammatory activity, whereas M2-like macrophages depend on oxidative phosphorylation to accomplish anti-inflammatory and tissue-repairing functions (16, 17). Understanding the mechanisms by which miR-210 influences macrophage phenotype can elucidate the complex balance between pro- and anti-inflammatory signals, which is essential for effective tissue repair and resolution of chronic inflammatory states. Therapeutic modulation of miR-210 might therefore represent a novel approach to fine-tune macrophage responses, potentially improving outcomes in conditions characterized by persistent inflammation or impaired tissue healing.

MiR-210 was recently reported to act as a molecular switch of macrophages toward the M1-like pro-inflammatory state, by reducing mitochondrial respiration in favor of glycolysis, thus modulating the macrophage metabolism and inflammatory response (15). Dysregulation of the inflammatory response drives persistent inflammation that manifests across diverse conditions, such as sepsis and diabetes, highlighting the critical need to maintain proper miR-210 levels (5, 18). However, the contribution of miR-210 to alternative (M2-like) macrophage polarization remains incompletely defined. In this study, the impact of miR-210 deletion on macrophages was examined, with a particular focus on the baseline transcriptome of resting macrophages and their capacity to adopt an IL-4-driven M2-phenotype. We report here that the deletion of miR-210 skews resting macrophages toward a pro-inflammatory transcriptional state, impairs the IL-4 induced polarization, and alters metabolic pathways essential for alternative M2 activation. Our findings highlight the pleiotropic and context-dependent functions of miR-210 in macrophage activation. Exploration of miR-210’s role in macrophage polarization not only advances our fundamental understanding of macrophage biology, but also opens new avenues for targeted therapeutic strategies.

## 2 Materials and methods

### 2.1 Mice

Animal experiments were conducted in accordance with the European Guidelines for Animal Welfare (Directive 2010/63/EU) and approved by the National Sanitary Veterinary and Food Safety Authority (389/22.03.2018 and 612/24.03.2021). C57BL/6J mice having a heterozygous deletion in miR-210 were a gift from Dr. Mircea Ivan (Indiana University School of Medicine, USA) and were obtained by crossing miR-210 floxed mice with Gata-1 Cre mice (19). MiR-210 knock-out (KO) and wild type (WT) controls were generated by breeding heterozygous mice. Mice were maintained under specific pathogen-free conditions in a controlled environment with a 12/12-h light/dark cycle, 21°C, and 55–60% humidity, and access to chow and water ad libitum.

### 2.2 Mouse genotyping

Tail tips obtained from mice at approximately 4 weeks of age were used for genomic DNA (gDNA) isolation, using the KAPA Mouse Genotyping Kit (Roche, KK7302) following the manufacturer’s instructions. PCR was performed using specific primers for deleted and non-deleted miR-210 and WT alleles and the PCR products were visualized by 1.5% agarose gel electrophoresis. The primer sequences are listed in **Table 1**.

**Table 1:** List of primer sequences designed for RT-qPCR.

Gene	Primer type	Sequence
Mir210_SA_F	Forward primer	ACACACATCTTTGAGGATCTATTGGGTCTG
Mir210_LA_R	Reverse primer	CTCTGAGTTTAATACCAGTGCCAGTCTAGA
Mir210_F	Forward primer	AGTGGAAAAGGATATCCAGGGAAGCTATAG
Mmd	Forward primer	GCATTCCTCATTGTTCCGGC
	Reverse primer	CCCATCCCGTAGATCCATGC
Gdf3	Forward primer	AGAGAAAGCGCCTTCACCTC
	Reverse primer	CACCCAGCTCCTTCACGTAG
Arg1	Forward primer	ACATTGGCTTGCGAGACGTA
	Reverse primer	ATCACCTTGCCAATCCCCAG
Rpl32	Forward primer	GTGGCTGCCATCTGTTTTACG
	Reverse primer	CGCCAGTTTCGCTTAATTTTCAC
Rab4a	Forward primer	AGAAGGACTTGGATGCCGAC
	Reverse primer	AAGCCTCTTCGACGTTCTCG
Pira2	Forward primer	CAGAAGCCAGCAAACAAGGC
	Reverse primer	GAAAGGCTGGGTGTCCAGTA
CD206	Forward primer	AACCACCACTGACTACGACAAA
	Reverse primer	AATCTCTCGCTTCCCTCAAAGTG
Retnl	Reverse primer	GTCAGCACAGACCTCTCTCTT
	Forward primer	GGGATGACTGCTACTGGGTG

### 2.3 Isolation of bone marrow cells

Long bones were extracted from hind limbs under sterile conditions. Bone marrow aspirates were obtained using the protocol previously described (20). Briefly, medium-sized channels were flushed with 5 ml of medium (high glucose DMEM medium supplemented with 10% fetal bovine serum) using a 25-G needle connected to a 5-ml syringe. The single-cell suspension obtained by passing the aspirate through needles of decreasing sizes (18G, 21G, 23G, 25G) was subsequently resuspended to a concentration of 10^6^ cells/ml and used for further experiments.

### 2.4 Monocyte purification

Monocytes were obtained using the EasySep™ Mouse Monocyte Isolation Kit (STEMCELL Technologies, #19761) following the manufacturer’s instructions. Briefly, approximately 60x10^6^ cells obtained from bone marrow aspirate were resuspended in 1 ml of EasySep buffer (STEMCELL Technologies, 20144) and incubated with an antibody cocktail targeting non-monocytic cells, including erythrocytes, lymphocytes, neutrophils, and dendritic cells. Afterward, EasySep™ Dextran RapidSpheres™ were added to the cell suspension to bind the antibody-labeled cells. The suspension was then placed into an EasySep™ magnet, which selectively retained the magnetically labeled cells while allowing the unmarked monocytes to be transferred into a new tube. The isolated monocytes were washed, counted, and either used for flow cytometry analysis or cultured *in vitro* to differentiate into macrophages for further experiments.

### 2.5 Macrophage differentiation and polarization

Isolated monocytes were seeded at 40,000 cells/cm² in differentiation medium consisting of high-glucose DMEM supplemented with 20% fetal bovine serum (FBS) and 30% L929-conditioned medium (L929-CM), serving as an endogenous source of M-CSF (21, 22). L929-CM was obtained from the supernatant of confluent L929 cells by sequential centrifugations at 400 × g for 5 minutes and 2,000 × g for 25 minutes. After 7 days in culture, with a medium refresh on day 3, polarization toward the M2 anti-inflammatory phenotype was induced by incubating the cells with 20 ng/mL IL-4 (R&D Systems, #RD-404-ML-010) for 72 hours. Control wells were maintained under identical conditions without IL-4 treatment.

### 2.6 RNA isolation and real-time PCR analysis

Total RNA was isolated from cells using the standard protocol of TRIzol reagent (Thermo Fisher Scientific, 15596026). One microgram of total RNA was used for reverse transcription, using the High-Capacity cDNA Reverse Transcription Kit (Thermo Fisher Scientific, MA, USA#4387406). RT-PCR was performed on a ViiA™ 7 Real-Time PCR System (Thermo Fisher Scientific) using SYBR™ Select Master Mix (Applied Biosystems, 4472918) or LightCycler^®^480 II (Roche) using Platinum^®^ Taq DNA Polymerase (Thermo Fisher Scientific, #10966-050). The relative expression level was determined using the comparative C_T_ method, with *Rpl32* used for normalization. MiR-210 expression levels were measured using TaqMan™ MicroRNA Assays (ThermoFisher Scientific, #A25576) in combination with the TaqMan™ MicroRNA Reverse Transcription Kit (ThermoFisher Scientific, #4366596). The expression of U6 snRNA was used as an endogenous control for normalization.

### 2.7 Fluorescence microscopy

Macrophages were washed twice with phosphate-buffered saline (PBS), fixed for 10 minutes with 4% PFA in PBS at room temperature, permeabilized for 10 minutes with 0.05% Triton X-100 in PBS, and blocked with 5% bovine serum albumin (BSA) in PBS for 1 hour at room temperature. Cells were then incubated overnight at 4°C with rat anti-mouse CD68 antibody (BioRad, MCA1957GA, 1:100) or rabbit anti-mouse alpha smooth muscle actin (αSMA) antibody (Abcam, Ab5694, 1:200), or 1 hour at room temperature with Alexa Fluor^®^ 647-labeled rat anti-mouse CD206 antibody (Biolegend, 141712, 1:200). After primary antibody incubation, cells were washed with PBS and incubated for 1 hour at room temperature with secondary antibodies: goat anti-rat Alexa Fluor ^®^ 488 (Invitrogen, A-11006, 1:250), or donkey anti-rabbit Alexa Fluor ^®^ 488 (Invitrogen, A-21207, 1:400). Nuclei were counterstained with 2 µg/ml Hoechst 22358 for 10 minutes. Cells were mounted with coverslips in Fluoromount-G™ Mounting Medium (Invitrogen, 00-4958-02), and imaged using a Leica DMi8 inverted fluorescent microscope with HC PL APO 10x/0.45 NA dry, and HC PL APO 40x/1.3 NA oil objectives. Fluorophores were excited with a multi-LED Spectra-X light source (Lumencor) and images were captured with a sCMOS camera Leica DFC9000 and subsequently processed with Leica LAS X software.

### 2.8 Metabolic profile analysis

The metabolic profile analysis was performed using the Agilent Seahorse XFp Cell Energy Phenotype Test Kit (Agilent, 103275-100), and Agilent Seahorse XFp Glycolytic Rate Assay (Agilent, 103346-100), following the general manufacturer’s instructions. Briefly, 25x103 cells in 200 µl differentiation medium were seeded into each well and allowed to adhere at 37°C under 5% CO2. After 24 hours, the medium was replaced with assay medium, followed by a 1-hour incubation at 37°C in a CO2-free environment. The analysis was performed using 1 µM oligomycin, 1 µM carbonyl cyanide-4 (trifluoromethoxy) phenylhydrazone (FCCP), 0.5 µM rotenone, and antimycin (Rot/AA) as stress factors.

### 2.9 Proliferation analysis

XTT assay (ThermoFisher Scientific, X6493) was performed to assess cell viability, according to the manufacturer’s guidelines. Briefly, M0 macrophages were seeded in 96-well plates and incubated in the presence or absence of IL-4. After three days, the culture medium was removed and fresh medium containing XTT reagent (1 mg/mL) supplemented with 25 µM phenazine methosulfate (PMS) was added to each well. The plates were incubated at 37°C under 5% CO_2_ atmosphere for 2 hours, allowing mitochondrial dehydrogenases to reduce XTT into its soluble formazan derivative. Formazan was quantified by reading the absorbance at 450 nm, on a microplate reader (Tecan Infinite M200 Multi-Detection Plate Reader). Cell proliferation was estimated in miR-210 KO cells in comparison to WT cells. Another method to assess cell proliferation was using nuclear staining with DAPI and flow cytometry analysis. Trypsinized cells were fixed in PBS with 4% PFA (paraformaldehyde) for 15 minutes at room temperature (RT), followed by permeabilization with 90% methanol on ice for 30 minutes. Next, nuclear DNA was stained with 1 µg/mL DAPI for 15 minutes at RT. Finally, the cells were resuspended in FACS buffer and analyzed by flow cytometry. Data were acquired on CytoFlex flow cytometer (Beckman Coulter) and analyzed using CytExpert software (Beckman Coulter).

### 2.10 Flow-cytometry analysis

Trypsinized cells were resuspended at 10^6^ cells/ml in FACS buffer (PBS + 2% FBS), and 100 μl of cell suspension was used for each sample. Cells were incubated with a mix of fluorescently labeled primary antibodies or corresponding isotype controls for 30 minutes on ice, followed by washing in FACS buffer. 2 µg/ml propidium iodide (PI) was added before analysis. At least 20,000 events were recorded for each sample, using a CytoFLEX Flow Cytometer (Beckman Coulter, U.S.A.) and the acquired data were analyzed using CytExpert version 2.5 software.

### 2.11 Phagocytosis assay

The phagocytic capacity of macrophages was assessed using pHrodo™ labeled S. aureus particles (Thermo Fisher, # P35367). These particles are engulfed by cells and emit a fluorescent signal when reached the acidic lysosomal compartment. Briefly, macrophages were seeded in 96-well plates (105 cells/well) and allowed to adhere for 2 hours at 37°C, and 5% CO2. Next, various amounts of pHrodo™ conjugate (0.1, 1, and 10 µg/µl) were added to the wells and incubated for 2 hours. Afterwards, the cells were washed and analyzed by flow cytometry. At least 20,000 events were recorded for each sample.

### 2.12 Efferocytosis assay

Murine macrophages were plated in 24-well plates at 1 × 10^5^ cells/cm² and allowed to adhere for 3 h at 37°C in complete medium. Jurkat cells serving as apoptotic targets were labelled with CellTracker™ Red CMTPX (3 µM in DMSO, 30 min, 37°C in serum-free RPMI), washed, and equilibrated for a further 30 min in complete RPMI medium. Apoptosis was then induced with 1 µM staurosporine for 3 h at 37°C, after which cells were rinsed twice in PBS. Labelled apoptotic Jurkat cells were added to the adherent macrophages at a 3:1 target-to-effector ratio and co-cultured for 2 h at 37°C. Non-engulfed Jurkat cells were removed by gentle PBS washing, and macrophages were detached and subjected to flow-cytometric analysis to quantify CMTPX signal.

### 2.13 Apoptosis assay

Murine macrophages were seeded at a density of 4 × 10^4^ cells/cm². After overnight adherence, cells were stimulated with 40 ng/mL recombinant murine TNF-α (R&D Systems 410-MT-025) for 24 hours in complete medium. Following stimulation, cells were gently detached and washed twice with PBS. Apoptosis was quantified using the Annexin V staining (BioLegend, 640906), following the manufacturer’s instructions. Briefly, cells were resuspended in Annexin V Binding Buffer at a density of 10^6^ cells/mL, and incubated with Annexin V-FITC and PI for 15 minutes at room temperature in the dark. Cells stained with Annexin V and PI were analyzed by flow cytometry.

### 2.14 ELISA

The level of cytokines in the supernatants of M0 and M2 macrophages was determined using the DuoSet ELISA kits (for IL-6, R&D Systems, DY406), IL-1β (R&D Systems, DY401-05), and the ELISA MAX™ Deluxe Set Mouse TNFα (BioLegend, 430904), according to the manufacturer’s instructions. Briefly, the 96-well plate wells were coated with the capture antibody overnight, followed by blocking with 1% BSA in PBS for one hour. The samples and standards were incubated for 2 hours at room temperature, followed by detection with a biotinylated antibody and streptavidin-HRP. HRP reaction was performed using TMB (3,3’,5,5’ tetramethylbenzidine) as the substrate. Reactions were stopped with 2 N H_2_SO_4_ and signal development was determined by measuring the absorbance at 450 nm with correction at 540/570 nm on a microplate reader (Tecan Infinite M200 Multi-Detection Plate Reader). Concentrations were determined using standard curves generated with recombinant cytokines.

### 2.15 Western blot analysis

Cell lysates were prepared by harvesting cells in 2x Laemmli Buffer. Equal volumes of samples (20 μl) were loaded onto 10% SDS-polyacrylamide gel and transferred onto nitrocellulose membranes using a semi-dry transfer system at 25 V for 30 minutes. The membranes were blocked with 1% fish gelatine in TBST (Tris buffered saline containing 0.1% Tween20) for 60 minutes at RT on a shaker. The membranes were incubated overnight at 4°C with antibodies against p53 (Abcam, #ab90363, 1:1000), TGF-β (Abcam, #ab215715, 1:1000), GAPDH (Abcam, #ab37168, 1:1000), IL-1β (Abcam, ab9722, 1:1000), αTubulin (R&D Systems, MAB9344 1:1000), β-actin (Sigma-Aldrich, A2228), and MMP-9 antibody (Novus Biologicals, NBP1-57840), prepared in 5% BSA in TBST. Washed membranes were then incubated with HRP-conjugated secondary antibodies for 1 hour at room temperature. Protein detection was performed using SuperSignal™ West Pico PLUS Chemiluminescent Substrate (Thermo Fisher Scientific, 34580) and visualized with a gel documentation system (ImageQuant LAS 4000 system, Fujifilm). Quantification was conducted using TotalLab™ software.

### 2.16 RNA sequencing and data analysis

For transcriptomic analysis, WT and KO macrophages under both M0 and M2 conditions were collected and lysed for total RNA extraction with TRIzol reagent (Thermo Fisher Scientific, 15596026) following the manufacturer’s protocol. RNA-seq was performed by Novogene (UK) Company Limited, following standard protocols. Library preparation included ribosomal RNA depletion, RNA fragmentation, and cDNA synthesis, followed by end repair, poly-A tailing, and adaptor ligation. The libraries were sequenced as stranded paired end reads on the Illumina NovaSeq 6000 platform. Initial quality control of raw sequencing reads was performed using FastQC (v0.12.0). Adapter sequences were trimmed with Trimmomatic (v0.39), and PCR duplicates were removed using Picard tools. Cleaned reads were aligned to the mouse reference genome (GRCm39 P110) using splice-aware STAR aligner (v2.7.11) (23). Alignment quality was evaluated with Samtools (v1.18) and stranded read quantification was performed using featureCounts (v2.0.3). Differential gene expression analysis was carried out with DESeq2 (v1.44.0) (24), using Wald test for two-group comparisons and applying the Benjamini-Hochberg procedure to account for multiple testing. Gene annotation was performed using BioMart, accessed via the Ensembl Genome Browser (release 112).

### 2.17 Enrichment analysis and visualization

Functional enrichment analysis of differentially expressed gene (DEGs) was conducted using DAVID (Database for Annotation, Visualization, and Integrated Discovery) for Gene Ontology (GO) Biological Processes (25) to compare the efficacy of polarization (DEGs for miR-210 KO_M2 vs miR-210 KO_M0 and WT_M2 vs WT_M0). Enrichr tool (https://maayanlab.cloud/Enrichr/) and R package fgsea (26) were used to identify significantly overrepresented biological pathways (27) and Molecular Signatures Database (MSigDB Hallmark 2020) was used as a reference to identify pathways impacted by miR-210 deletion, in both phenotypes. Overlapping DEGs between conditions were visualized using Venn diagrams. Data visualization included heatmaps, PCA plots, and volcano plots, generated using the ggplot2 package in R (28) and GraphPad Prism 8.0.1 (GraphPad Software, San Diego, CA, USA).

### 2.18 Statistical analyses

Data were analyzed using GraphPad Prism 8.0.1 (GraphPad Software, San Diego, CA, USA). The results are expressed as mean ± standard error of the mean (SEM). The mean values were obtained from at least three independent experiments. Data analysis included Student t-test`s and multiple t-tests for comparisons between mir-210 KO *vs*. WT cells within the same phenotype, and two-way ANOVA to evaluate the effects of genotype (WT *vs*. KO) and polarization (M0 *vs*. M2) on gene expression. A p value less than 0.05 was considered statistically significant.

## 3 Results

### 3.1 Characterization of macrophages obtained from WT and miR-210 KO mice

**Figure 1:**
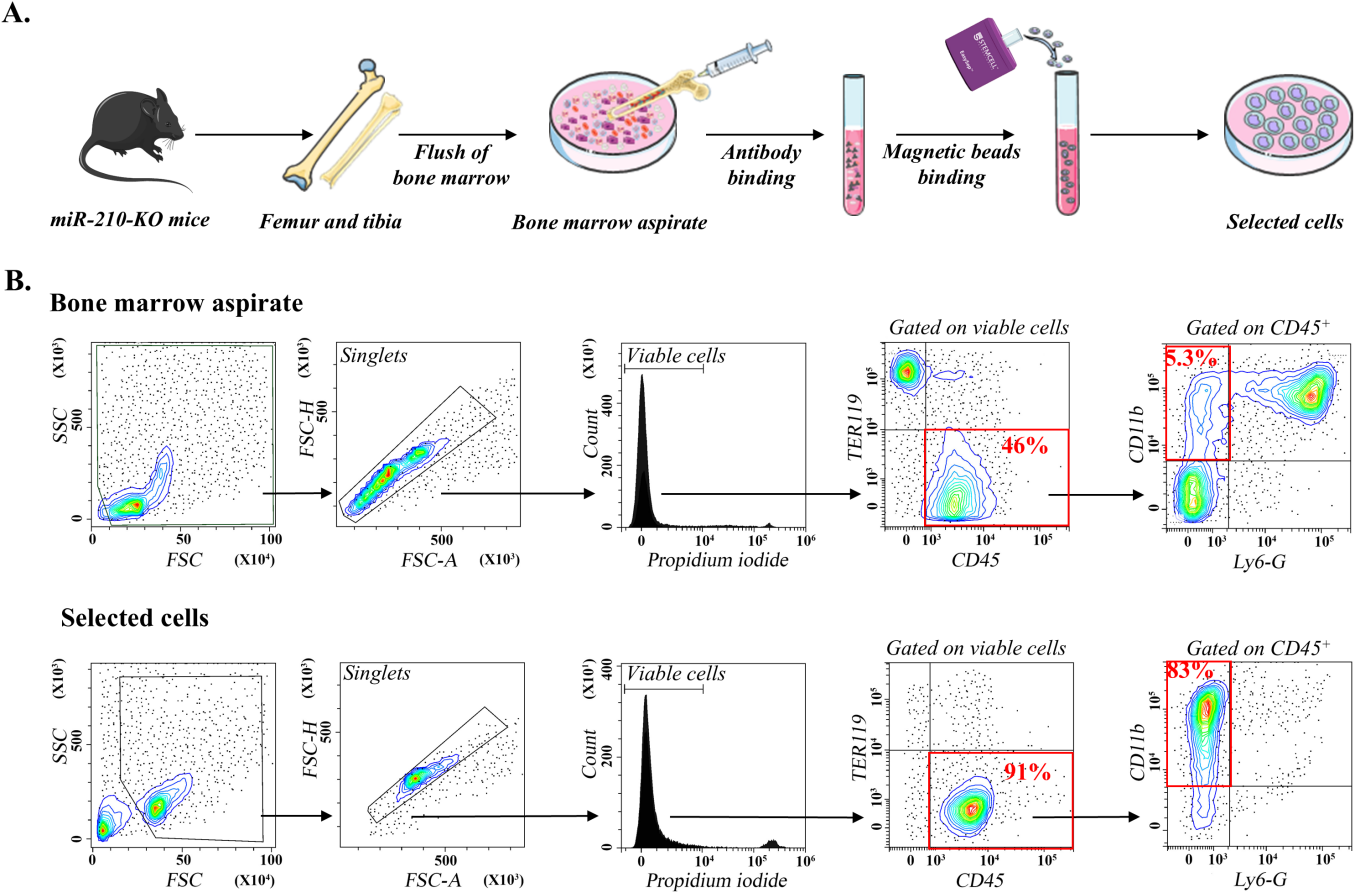
Isolation and differentiation of miR-210-KO bone marrow monocytes. **(A)** Schematic representation of the isolation and selection of bone marrow monocytes from miR-210 KO mice. **(B)** Flow cytometry analysis of bone marrow aspirates (upper) and selected cells (lower). Note the enrichment in CD11b^+^ cells after selection. The flow cytometry plots are representative of at least three independent experiments performed using bone marrow cells from WT and miR-210-KO mice. The data illustrated correspond to one miR-210 KO mouse. No differences were observed between WT and KO animals regarding the initial monocyte populations or the efficiency of the negative selection procedure.

MiR-210 KO and WT mice were derived from heterozygous (miR-210^+^/^−^, HET) matings. Genotypes were confirmed by PCR (**Supplementary Figure S1A**). Bone marrow-derived monocytes from WT and miR-210 KO mice were isolated using a magnetic bead-based purification approach (**Figure 1A**), which was considered advantageous over flow cytometry-based sorting, as it leaves target cells untouched, thereby preserving their native phenotype and functional integrity. Importantly, the enrichment process increased the purity of CD11b^+^ cells (**Supplementary Figure S1B**) from approximately 25% in BM cells (which included the neutrophil population) to over 75% in the selected cells after neutrophil depletion, while fully preserving cell viability (PI staining, **Figure 1B**), indicating minimal cell damage during isolation. Flow cytometry analysis validated the enrichment process, demonstrating effective depletion of non-monocyte populations, including erythrocytes (TER119^+^ cells) and neutrophils (CD11b^+^/Ly-6G^+^ cells), while yielding a monocyte-enriched (CD11b^+^/F4/80^−^) cell fraction (**Figure 1B**). No differences were observed between WT and KO animals regarding the phenotype of the initial monocyte populations or the efficiency of the negative selection procedure (data not shown). To further ensure the maintenance of physiological relevance, purified monocytes were immediately cultured following isolation.

**Figure 2:**
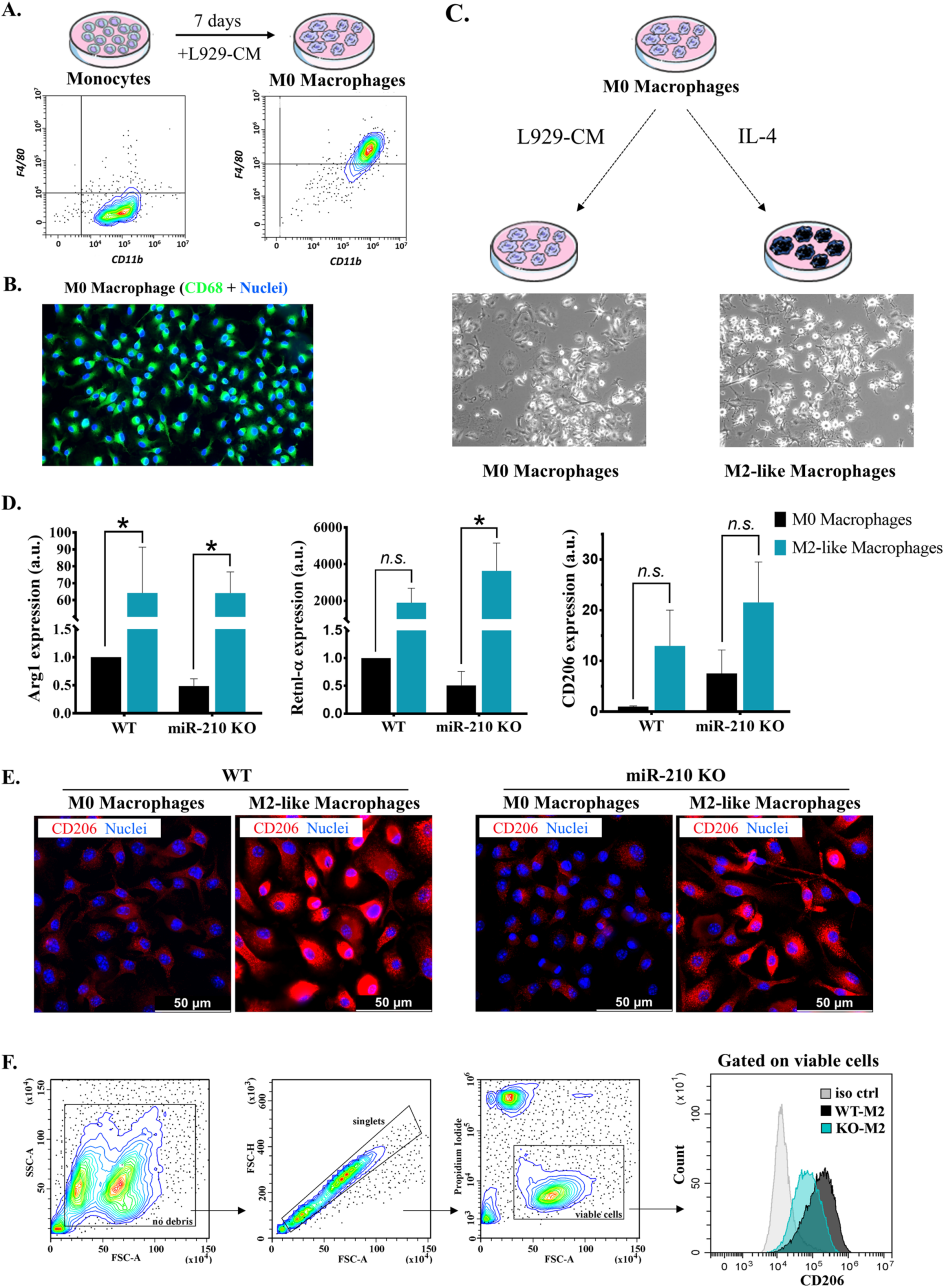
Characterization of M0 and M2-like macrophages. **(A)** Flow-cytometry characterization of monocytes and M0 macrophages, showing the gain of F4/80 marker after differentiation. **(B)** Immunofluorescence staining showing the presence of CD68 (green). Hoechst was used for nuclear counterstaining. **(C)** Schematic representation of macrophage polarization towards M2-like phenotype. Representative phase-contrast images of M0 and M2-like cells. **(D)** Gene expression analysis of M2 macrophage polarization markers in WT and miR-210 KO macrophages. Data are presented as mean ± SEM from 3-5 animals. Statistical significance was determined using two-way ANOVA, *p < 0.05, *n.s*= not significant. **(E)** Representative immunofluorescence images showing CD206 (red) and nuclei (blue, Hoechst) in WT and miR-210 KO macrophages in both M0 and M2 polarization states. Note the increased CD206 signal in WT M2 macrophages compared to KO M2 macrophages. Scale bar = 50 μm. **(F)** Flow cytometry analysis of CD206 surface expression in M2 macrophages. The gating strategy is shown on the left. The histogram (right) displays comparative CD206 levels in WT-M2 and KO-M2 macrophages. Note a higher signal in WT-M2 macrophages vs. KO-M2.

Purified monocytes differentiated in L929-CM for seven days yielded a homogenous CD11b^+^, F4/80^+^ M0-resting macrophage population (**Figure 2A**). Differentiated cells expressed the pan-macrophage marker CD68, as illustrated by fluorescence microscopy (**Figure 2B**). Despite the confirmed absence of miR-210 in M0 macrophages (**Supplementary Figure S2A**), no differences were noted between miR-210 KO-derived and WT cells regarding CD68 and F4/80 marker expressions (data not shown), underscoring a similar differentiation trajectory. Additionally, flow cytometry analysis demonstrated the phagocytic capacity of differentiated cells, through their capacity to engulf bacterial particles, a particular feature of macrophages (**Supplementary Figure S2B**). However, a slight yet significant difference was observed in M0 cell size and internal complexity, with miR-210 KO cells displaying lower side scatter (SSC) values compared to WT cells (**Supplementary Figure S2C**). This reduction in granularity likely reflects minor alterations in organelle and vesicle content in miR210 KO cells (29) rather than impaired differentiation.

To assess macrophage polarization towards a M2-like phenotype, M0 macrophages were stimulated with IL-4 for three days (**Figure 2C**). Real-time PCR analysis confirmed successful polarization, as evidenced by the upregulation of *Arg1*, *Retnl-α*, and *CD206* genes (**Figure 2D**). Notably, a higher upregulation of CD206 was observed in WT-derived M2-like cells (compared to those from miR-210 KO mice, although the difference did one did reach statistical significance due to high variability in fold change values. A similar trend was observed at the protein level by immunocytochemistry (**Figure 2E**) and further supported by flow cytometry analysis (**Figure 2F**), which confirmed higher *CD206* expression in M2-like macrophages derived from WT compared to miR-210 KO mice. Together, these data suggest that although miR-210 KO-derived macrophages are capable of differentiating into M2-like cells *in vitro*, the extent of polarization appears to be slightly reduced compared to WT cells.

### 3.2 Transcriptomic profiles of miR-210 KO and WT- derived macrophages

**Figure 3:**
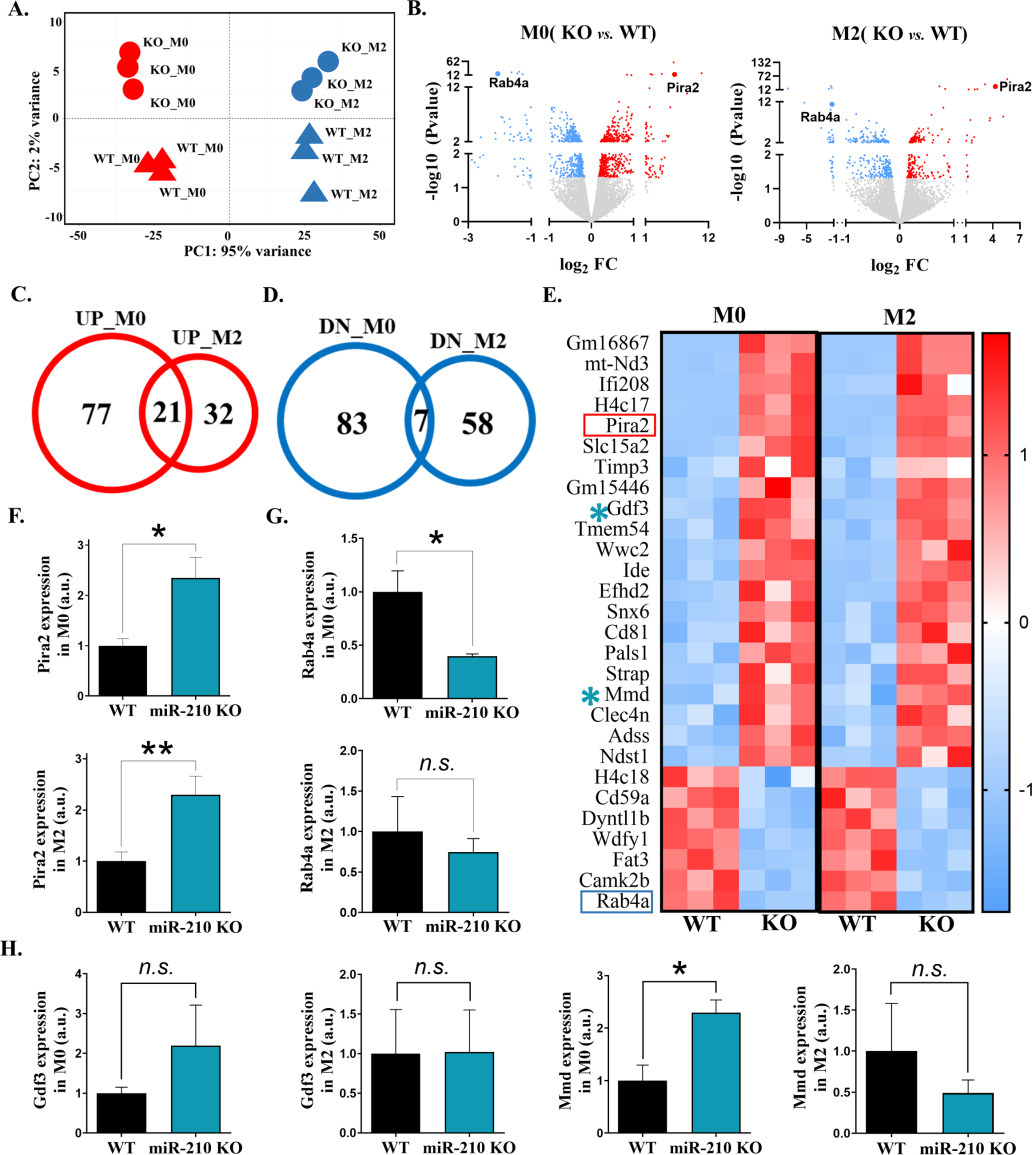
Transcriptomic analysis of miR-210 KO macrophages. **(A)** PCA representation, showing clustering of WT and miR-210 KO macrophages under M0 and M2 conditions based on RNA-seq data performed with R software. **(B)** Volcano plots showing significantly upregulated (red) and downregulated (blue) genes in M0 (left) and M2 (right) KO macrophages relative to WT controls. **(C, D)** Venn diagrams showing the overlap of upregulated **(C)** and downregulated **(D)** genes in M0 and M2 macrophages in KO versus WT cells. **(E)** Heatmap of the 28 common DEGs identified in the Venn diagrams. **(F, G)** RT-qPCR validation of *Pira2* **(F)** and *Rab4*a **(G)** expressions in M0 (upper) and M2 (lower) macrophages. Data are presented as mean ± SEM, of n=6 **(F)** or n=3 **(G)** experiments. Statistical significance was determined using Student t-test, *p < 0.05, **p < 0.01, *n.s*.= not significant; **(H)** qRT-PCR analysis of *Gdf3* and *Mmd* expressions in M0 and M2 macrophages. Data are presented as mean ± SEM from 6 experiments. Statistical significance was determined using Student’s t-test, *p < 0.05, *n.s*.= not significant.

To assess the impact of miR-210 deletion on macrophage transcriptomic signature, RNA sequencing was performed on M0 and M2 macrophages derived from WT and miR-210 KO mice. Principal Component Analysis (PCA) demonstrated distinct clustering patterns based on polarization state (M0 vs. M2) and genotype (miR-210 KO vs. WT). PCA resolved four discrete clusters (**Figure 3A**): PC1, explaining 95% of total variance, separated M0 from M2 samples, whereas PC2, accounting for 2%, distinguished miR-210 KO from WT macrophages. Analysis of DEGs (FDR < 0.05, |log_2_FC| > 0.263) was conducted to identify transcriptional changes in miR-210 KO macrophages compared to WT controls. Volcano plots illustrated many deregulated genes in both M0 and M2 states (**Figure 3B**). To further characterize these changes, we identified consistently upregulated or downregulated genes overlapping in M0 and M2 phenotypes, highlighting shared transcriptional responses to miR-210 deletion (**Figures 3C, D**). A heatmap visualization of overlapped DEGs was generated using the following cutoffs: average normalized counts >10, |log_2_FC| > 0.263, and FDR <0.05 (**Figure 3E**). Two of the most deregulated genes, *Pira2* and *Rab4a*, selected based on their high expression levels in the RNA-seq dataset, compared with other DEGs, were independently validated by qRT-PCR (**Figures 3F, G**).

To assess whether DEGs identified in M0 and M2 macrophages included direct miR-210 targets, we intersected genes that were upregulated upon miR-210 KO with predictions from three microRNA target resources (**Supplementary Figure S3A**): miRDB 6.0 (30), TargetScan 8.0 (31), and DIANA-microT 2023 (32). In M0 cells, predicted targets that were significantly upregulated included *Gsr* and *Txnrd1*, encoding reductases of glutathione and thioredoxin, Acetyl-CoA synthetase Acss2, and a transglutaminase Tgm2 involved in phagocytosis and differentiation (**Supplementary Figure S3B**, **Supplementary Table S1**). In M2 polarization state, upregulated genes included two encoding ion exchangers Clcn5 (H+/Cl-) and Slc9a9 (Na+/H+), and Zmat3, encoding for a p53-sensitive splicing regulator protein (**Supplementary Figure S3B**, **Supplementary Table S1**). *Gdf3* and *Mmd*, the only predicted targets shown to be significantly upregulated in both M0 and M2 states (**Supplementary Figure S3B**), were subjected to RT-qPCR validation using an independent experimental batch. Although the expression trends aligned with those observed in the sequencing data (**Supplementary Figure S3B**), statistical significance was not reached for Gdf3. In contrast, *Mmd* was significantly upregulated in miR-210 KO macrophages, with this increase observed only in M0 macrophages (**Figure 3H**).

### 3.3 Molecular consequences of miR-210 deletion in macrophages

**Figure 4:**
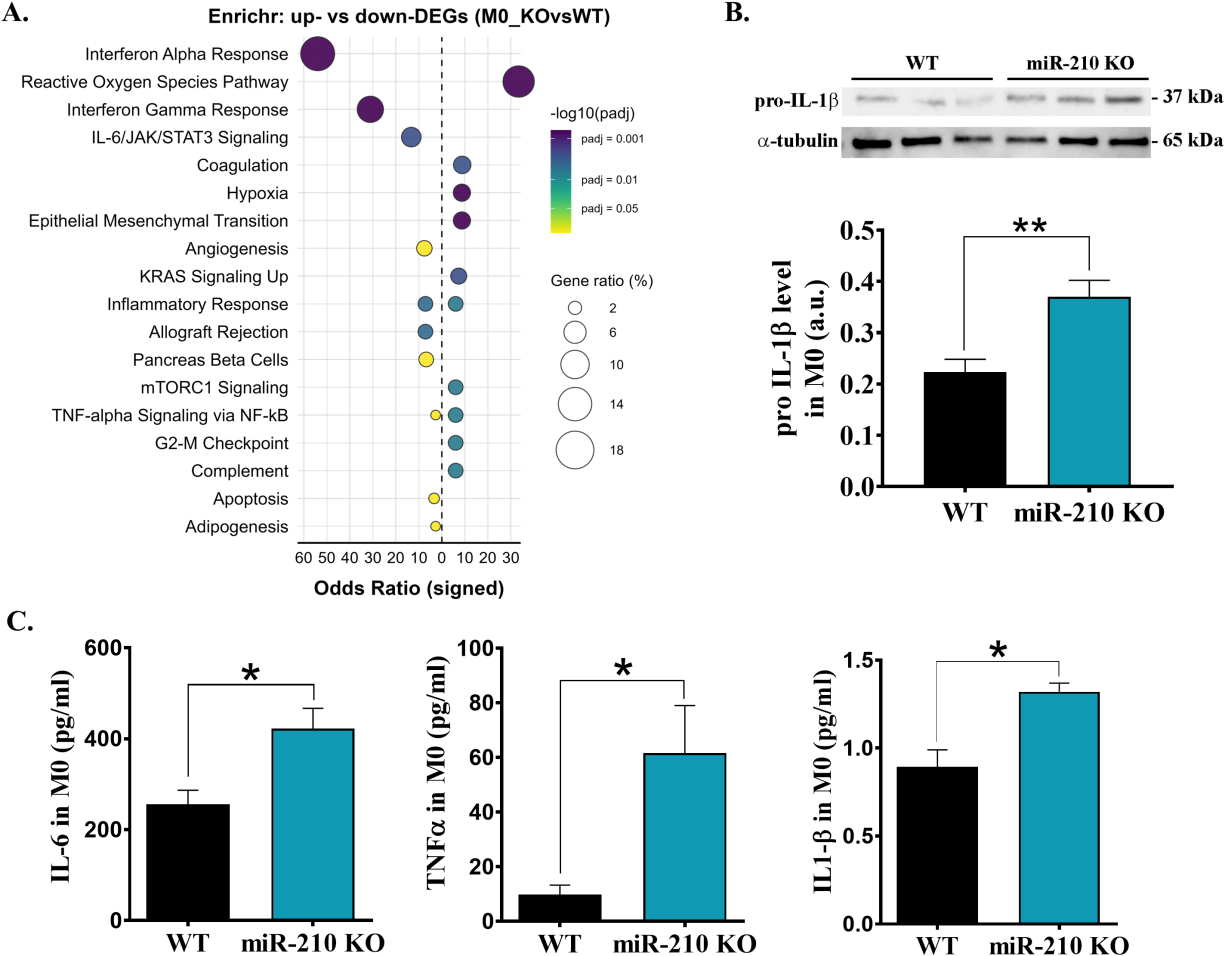
Effects of miR-210-KO in M0 macrophages. **(A)** Enrichr dot-plot representation for pathways enriched among differentially expressed genes in miR-210-KO versus WT M0 macrophages. The signed odds ratio (x-axis) indicates over-representation in up-regulated (positive) or down-regulated (negative) genes. **(B)** Western blot analysis of pro-IL1β levels in M0 macrophages, with α-tubulin used as a loading control. Densitometry analysis performed using TotalLab. Data are presented as the mean ± SEM, n = 3 mice per condition. Statistical significance was determined using Student’s t test, *p< 0.05. **(C)** ELISA quantification of IL-6, TNF-α and IL-1β in the supernatant of M0 macrophages. Data are presented as mean ± SEM, n = 3 for IL-6 and IL-1β and n=4 for TNF-α.

To investigate the molecular consequences of miR-210 deletion in macrophages, DEGs identified in both M0 and M2 macrophages were initially subjected to pathway overrepresentation analysis using the Enrichr tool. To this, we queried only the significant upregulated or downregulated DEGs against MSigDB Hallmark 2020 database. In miR-210 KO M0 macrophages the upregulated DEGs were primarily associated with reactive oxygen species signaling, hypoxia or pro-inflammatory pathways, while downregulated DEGs were linked to interferon alpha-gamma response and IL-6/JAK/STAT3 signaling (**Figure 4A**). Concordant with these signatures, pro-IL-1β protein and secreted IL-6, TNF-α and IL-1β levels were significantly higher in KO macrophages (**Figures 4B, C**). Together, these data indicate that miR-210 serves as a brake on basal inflammatory tone in resting M0 macrophages, restraining both cytokine gene programs and effector release.

**Figure 5:**
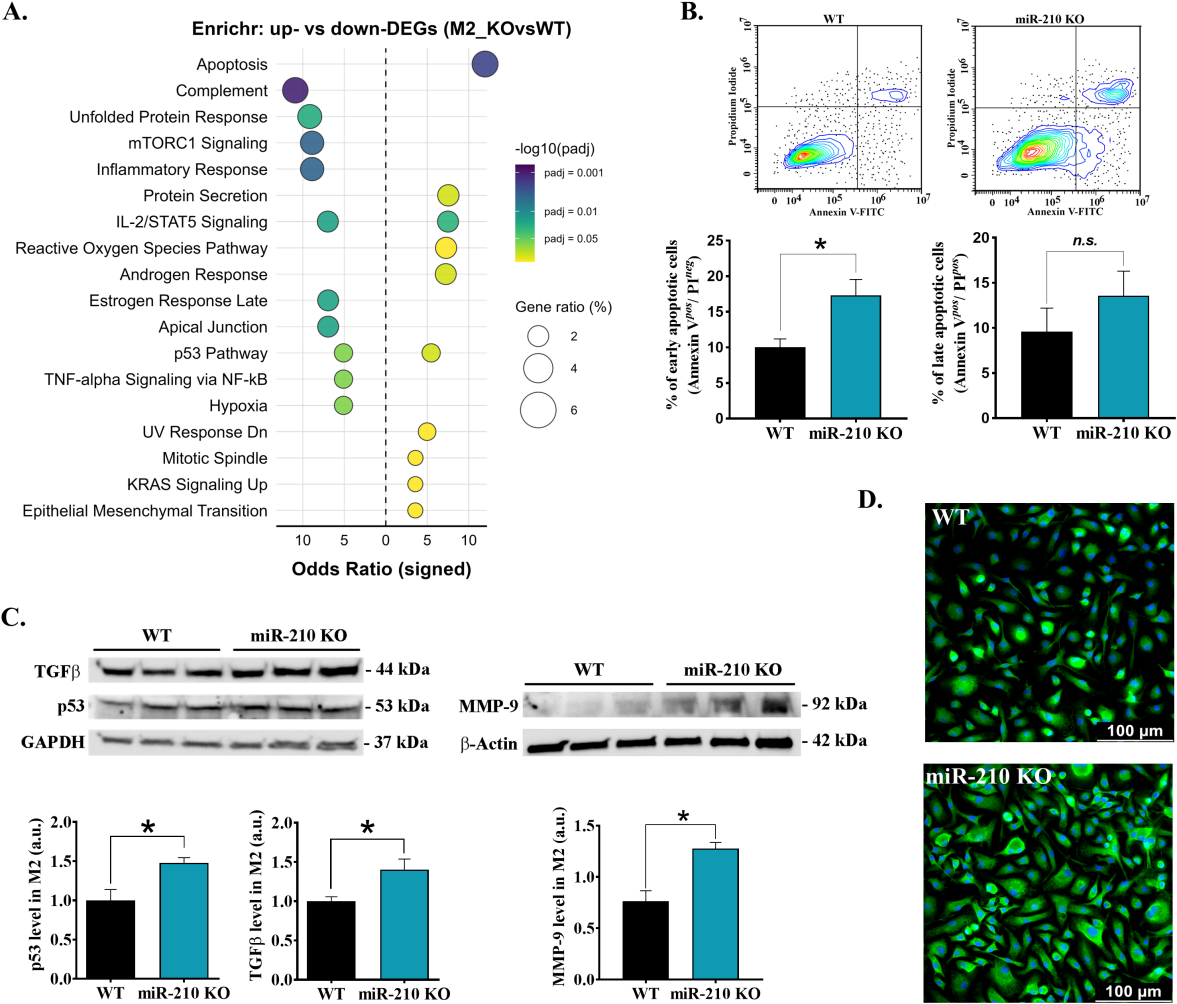
Effects of miR-210-KO in M2 macrophages. **(A)** Enrichr dot-plot representation for pathways enriched among differentially expressed genes in miR-210 KO versus WT M2 macrophages. **(B)** Apoptosis in M2 macrophages after 24 h exposure to TNF-α (40 ng/mL). (Top) Representative Annexin V/Propidium dot-plots and quantification early (Annexin V^+^/PI^-^) and late (Annexin V^+^/PI^+^) apoptotic cells (bottom). Data are presented as mean ± SEM (n = 3 mice per group). Statistical significance: Student’s t-test, *p < 0.05; *n.s.*, not significant. **(C)** Western blot analysis of TGF-β, p53, and MMP-9 levels in M2 macrophages. GAPDH and -actin were used as loading controls. Densitometry analysis performed using TotalLab. Data are presented as the mean ± SEM, n =6 (for TGF-β) and n = 3 (for p53 and MMP-9) mice per condition. Statistical significance was determined using Student’s t test, *p< 0.05. **(D)** αSMA immunofluorescence staining (green) in IL-4-polarized M2 macrophages showing a higher signal in miR-210 KO cells as compared to WT cells. Nuclei counter-stained with Hoechst (blue). Scale bar, 100 µm. Images are representative of n=3 mice per genotype.

**Figure 6:**
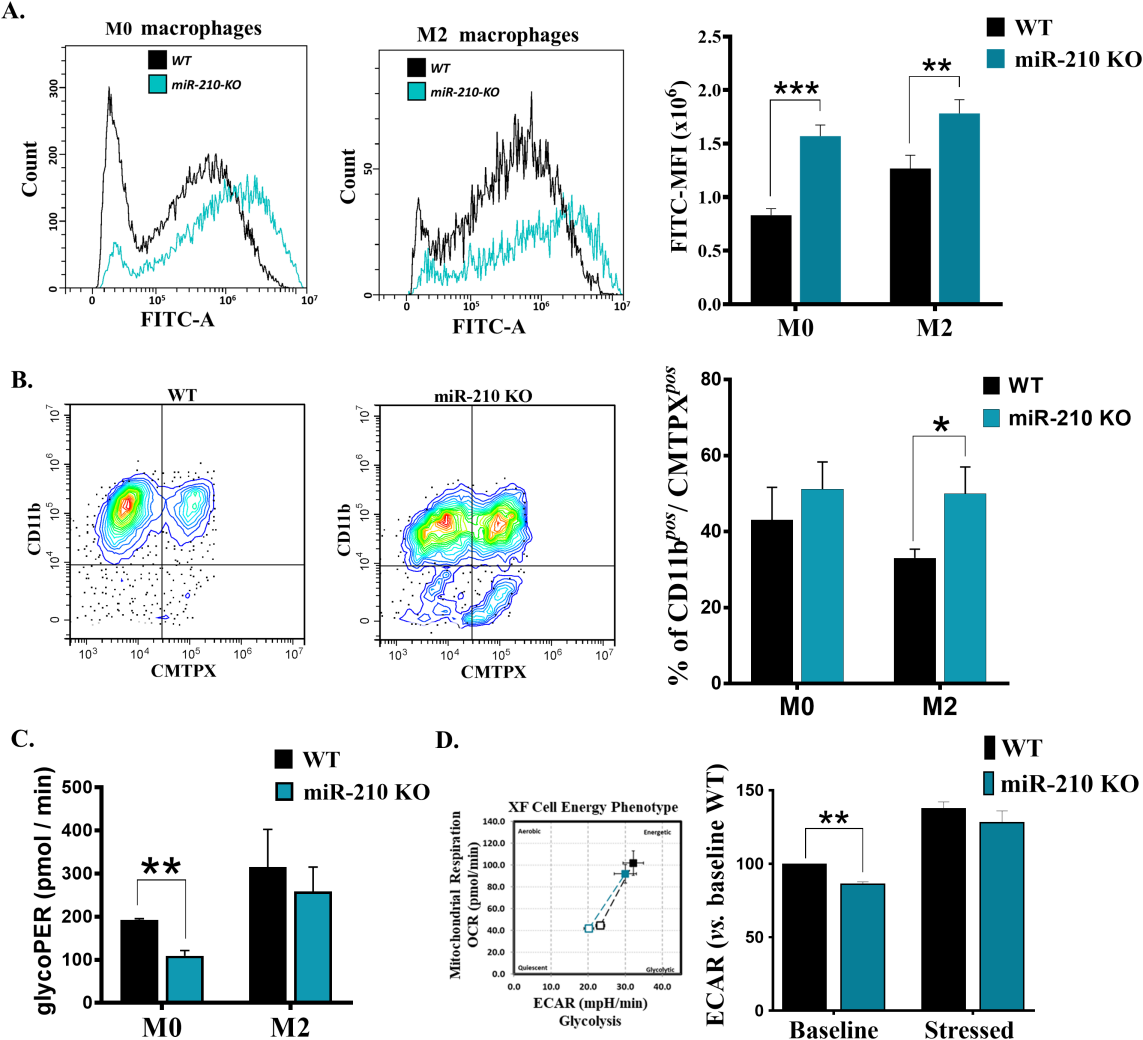
Functional and metabolic characterization of miR-210 KO macrophages. **(A)** Flow cytometry analysis of phagocytic capacity of M0 (left) and M2 (middle). (Right) Histogram showing the quantification of phagocytic capacity in M0 and M2 cells. MFI, Mean fluorescence intensity. Data are presented as mean ± SEM, n = 6 per condition. Statistical significance: two-way ANOVA, **p < 0.01, ***p < 0.005. **(B)** Flow-cytometry analysis illustrating the efferocytosis capacity of WT and miR-210 KO macrophages. (Left and middle panels) Representative contour plots showing CD11b vs. CMTPX fluorescence in WT and miR-210 KO M2 cells. (Right panel) Quantification of macrophages (CD11b^+^) that engulfed CMTPX+ apoptotic bodies (CD11b⁺ CMTPX^+^) expressed as percentage of total macrophages under M0 and M2 polarization conditions. Data are presented as mean ± SEM from three independent experiments. Statistical significance was determined using unpaired two-tailed Student’s t-test, *p < 0.05. **(C)** Seahorse Glycolytic Proton Efflux Rate (glycoPER) showing decreased glycolysis in miR-210 KO cells only in M0 phenotype. Data are presented as mean ± SEM, n = 3 per condition. Statistical significance was determined using unpaired Student-t test, **p < 0.01. **(D)** (Left) Representative image of a Seahorse XF Cell Energy Phenotype Analysis showing metabolic shifts in basal and stress conditions in WT and KO macrophages. (Right) Histogram representation of ECAR values. Data are presented as mean ± SEM, n = 3 per condition. Statistical significance was determined using Welch's t test, **p < 0.01.

Conversely, pathway enrichment analysis of M2 macrophages revealed apoptosis as the main pathway associated with upregulated DEGs, while mTORC1 signaling, inflammatory response, IL-2/STAT5 signaling, p53 pathway, and complement pathway were associated with downregulated DEGs in miR-210 KO macrophages (**Figure 5A**). These findings were further supported by a significant increase in apoptosis (**Figure 5B**) and in the protein level of p53, TGFβ, MMP-9 proteins in M2 macrophages from miR-210 KO-derived cells, as compared to WT cells (**Figure 5C**). Consistent with a stress-associated, fibrogenic shift, α-SMA protein was also overexpressed in KO macrophages (**Figure 6D**). Collectively, these observations indicate that miR-210 suppresses stress-response and fibrotic pathways in M2 macrophages, thereby preserving their reparative identity.

To validate the over-representation enrichment results, we subsequently applied rank-based GSEA to the full transcriptome providing a complementary, threshold-independent assessment of pathway perturbation (**Supplementary Figure S4**, **Supplementary Tables S2**, **S3**). In miR-210 KO M0 macrophages, GSEA analysis revealed broad up-regulation of cell-cycle checkpoint genes (e.g., *Stmn1*, *Mt2*, *H2ax*), metabolic programs (MYC targets, mTORC1, hypoxia, glycolysis, OXPHOS) and reactive-oxygen species pathways (*Gsr*, *Txnrd1*, *Srxn1*, *Gclm*, *Prdx6*, *Cat*) (**Supplementary Figures S4A, B**). By contrast, the type-I and type-II interferon responses were the only two pathways significantly down-regulated, driven by reduced expression of canonical ISGs (*Gbp2*, *Gbp4*, *Gbp9*, *Ifit1bl1*, *Ifi44*, *Isg15*) (**Supplementary Figures S4A, B**). In miR-210 KO M2 macrophages, GSEA showed coordinated suppression of pro-inflammatory and stress-response programs (**Supplementary Figures S4C, D**), including mTORC1 (*Trib3*, *Asns*), NF-κB/TNF-α signaling (*Zfp36*, *F3*), IFN-γ response (*Rnf31*, *Pim1*), and unfolded-protein response genes (*Psat1*, *Chac1*).

Collectively, these findings highlight a context-dependent role for miR-210: its loss promotes cell-cycle and metabolic re-wiring that favors a pro-inflammatory state in resting macrophages, whereas in M2 macrophages it dampens inflammatory signaling yet drives a pro-fibrotic, apoptosis-prone phenotype.

### 3.4 The impact of miR-210-deletion on the metabolic profile and phagocytic activity in macrophages

To translate the transcriptomic shifts into functional terms, we compared phagocytosis, efferocytosis, and glycolytic activity in WT and miR-210 KO macrophages. Comparative analysis of phagocytic activity, evaluated by the capacity of cells to engulf FITC-labeled Staphylococcus aureus, demonstrated an increase in FITC intensity in miR-210 KO macrophages compared to WT under both M0 and M2 conditions (**Figure 6A**). Conversely, miR-210 KO macrophages also displayed superior clearance of apoptotic Jurkat cells. The proportion of CD11b^+^ CMTPX^+^ double-positive cells rose significantly in miR-210 KO M2 cultures and showed a similar, non-significant trend in M0 cells (**Figure 6B**). Comparative determination of the glycolytic proton efflux rate (glycoPER) using Seahorse XF Glycolytic Rate Assay Kit showed a reduced level of basal glycolysis in miR-210 KO M0, but not in M2, macrophages compared to WT (**Figure 6C**). This reduction in basal glycolysis in miR-210 KO M0 macrophages was confirmed by a reduced ECAR in the basal state. Noteworthy, Seahorse XFp Cell Energy Phenotype Test showed the capacity of miR-210 KO cells to restore ECAR in the presence of stress factors (**Figure 6D**), thus suggesting the potential of KO cells to adapt the metabolic potential under stress conditions.

### 3.5 Role of miR-210 in M2 polarization

**Figure 7:**
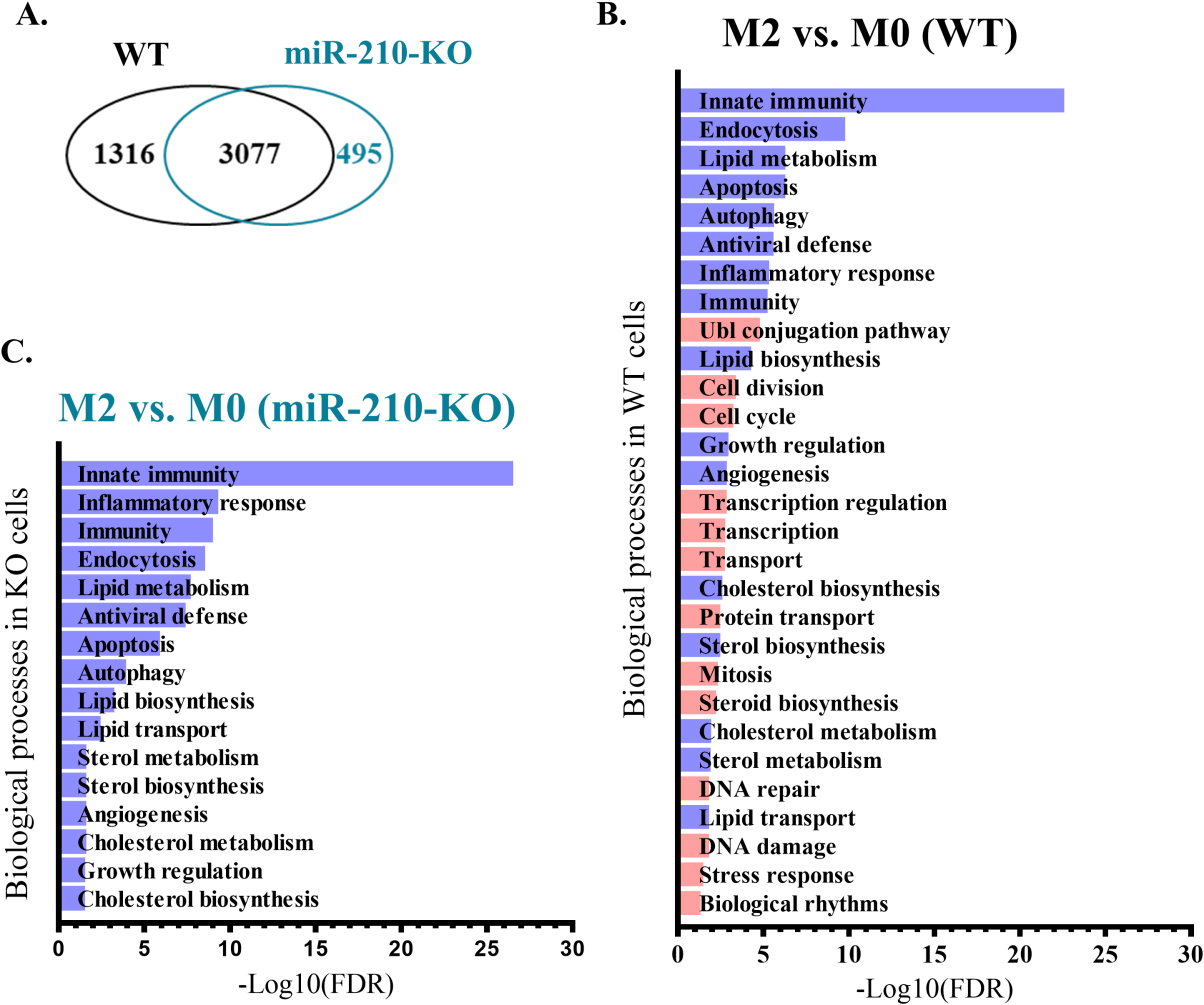
Biological processes modified during M2 polarization in WT and miR-210-KO M0 macrophages. **(A)** Venn diagram representing the overlapping DEGs during the polarization process. **(B, C)** Pathway enrichment analysis (performed using DAVID) of biological processes differentially regulated during M2 polarization in WT **(B)** and miR-210-KO **(C)** cells. Purple color indicates the common pathways impacted in both genotypes, and orange color indicates the unique pathways impacted only in the polarization process from WT mice.

**Figure 8:**
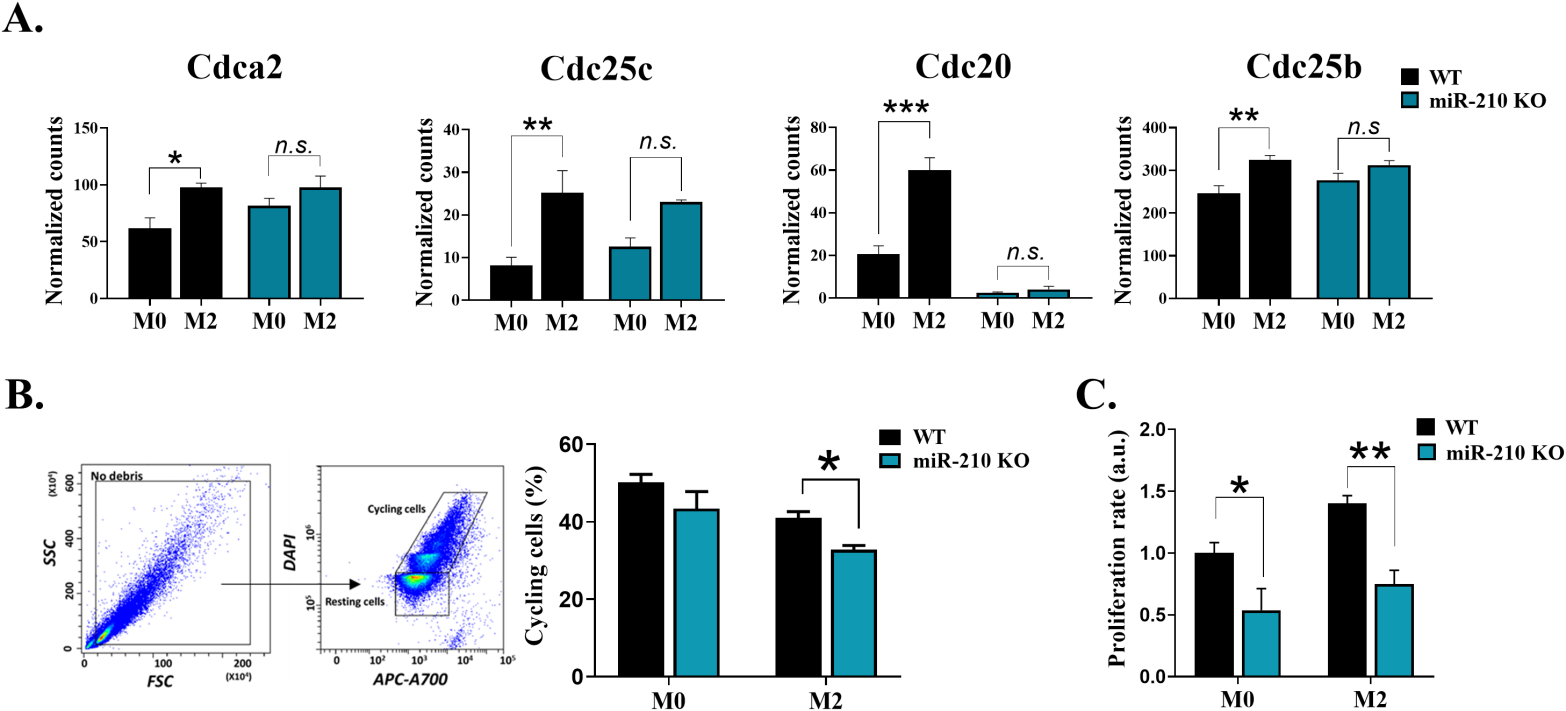
Impact of miR-210 deletion on macrophage cell cycle and proliferation. **(A)** Normalized counts of *Cdca2*, *Cdc20*, *Cdc25*, and *Cdc25b* obtained from RNA seq data in M0 and M2 macrophages. **(B)** Cell cycle analysis of M0 and M2-like macrophages from WT and miR-210 KO mice, using DAPI staining and flow cytometry. Representative dot plots showing the gating strategy for analysis. The bar graph illustrating the percentage of cycling cells, in M0 and M2 cells. Data are presented as mean ± SEM, from three independent experiments. Statistical significance was determined using Unpaired Student-t test, *p < 0.05, **p < 0.01, ***p < 0.005, *n.s.*, not significant. **(C)** Cell proliferation (XTT assay) of miR-210 KO and WT macrophages in M0 and M2 conditions. Data are presented as mean ± SEM from three independent experiments. Statistical significance was determined using two-way ANOVA, *p < 0.05, **p < 0.01.

To evaluate the impact of miR-210 deletion on macrophage polarization towards the M2-like phenotype, we focused on the protein-coding genes with significant changes during the M0 to M2 transition (average normalized counts >10, |log_2_FC| > 0.263, FDR<0.05). The analysis identified unique and overlapping sets of genes as illustrated in Venn diagram (**Figure 7A**). Gene ontology analysis demonstrated that the biological processes significantly impacted during miR-210 KO and WT macrophage polarization (FDR <0.05) were only partially overlapped, with those found in KO cells representing only a subset of those found in WT cells (**Figures 7B, C**). The M0-M2 transition is accompanied by activation of cell-cycle machinery, coupling phenotypic polarization with the proliferative expansion needed for effective tissue repair. Notably, our analysis showed that several biological processes, including cell division, cell cycle regulation, DNA repair, and DNA damage, were enriched only in WT cells during macrophage polarization while they did not reach statistical significance during the polarization process of miR-210 KO cells (**Figures 7B, C**, highlighted in orange). Indeed, RNA-seq data analysis revealed four transcripts of genes involved in cell cycle and division (*Cdca2, Cdc25c, Cdc20*, and *Cdc25b*) that showed significantly increased expression in WT cells during the transition from the M0 to M2 states but remained unaltered in miR-210 KO cells under the same experimental conditions (**Figure 8A**). Further support for this hypothesis was obtained through functional validation of cell proliferation by DAPI staining and flow cytometry, which revealed a lower proportion of cycling cells in miR-210 KO-derived M2 macrophages compared to WT (**Figure 8B**). Moreover, XTT assay data indicated a reduced proliferative rate in miR-210 KO macrophages compared to WT cells (**Figure 8C**). Overall, these findings indicate a potential role for miR-210 in cell cycle progression during macrophage polarization.

Collectively, these data suggest that miR-210 plays an important role in complete macrophage polarization towards the pro-reparatory phenotype, with potential implications for chronic inflammation, immune dysfunction, and tissue repair.

## 4 Discussion

The hypoxia-responsive miR-210 play a pivotal role in orchestrating cellular responses to oxygen deprivation (33). Under hypoxic conditions commonly present in sites of inflammation or injury, miR-210 is strongly upregulated and modulates macrophage activation through metabolic rewiring mediated by HIF-1α and NF-κB signaling (5, 34), and its function varies significantly depending on the polarization cues provided by the microenvironment. This metabolic shift is critically involved in determining macrophage functional phenotypes. (35, 36).

Recognizing the limited understanding of miR-210’s influence in M2-like activation, this study was designed to define the role of miR-210 in guiding macrophages toward an M2 phenotype, with a particular focus on cell energy phenotype, efferocytotic activity, and alternative polarization capacity. The novel findings of this study are: (i) miR-210 deletion skews resting macrophages toward a pro-inflammatory phenotype, characterized by altered expression of cytokines and surface markers; (ii) Polarization toward the M2-like phenotype is impaired in miR-210 KO macrophages, as evidenced by suboptimal transcriptional re-wiring; (iii) Cell cycle progression is disrupted in the absence of miR-210, with impaired upregulation of genes involved in cell cycle and reduced proliferation of M2 macrophages.

In this study, we employed monocytes isolated from mouse bone marrow using a negative selection kit that specifically removed unwanted cell populations without introducing external stimuli. The monocytes were subsequently differentiated *in vitro* into M0 resting macrophages, using L929-conditioned medium that provided the cells with M-CSF, a cytokine promoting monocyte differentiation into macrophages (21, 37). After 7 days in culture, macrophage identity was confirmed by the co-expression of CD11b and F4/80 (38, 39) and the presence of CD68, a cytoplasmic marker of macrophages (40). Functional assessment was determined through phagocytosis assays. The resting M0 macrophages were subsequently polarized toward an M2-like state in the presence of IL-4, and the confirmation of successful polarization by the upregulation of M2-specific markers, including *Arg1*, *CD206*, and *Retnl-α* (41).

Transcriptomics profiling in miR-210 KO-derived macrophages revealed several upregulated predicted miR-210 targets, consistent with a context-dependent loss of post-transcriptional regulation by miR-210. Specifically, in M0 macrophages, the increase of *Gsr* and *Txnrd1* suggests enhanced antioxidant cellular capacity via the glutathione and thioredoxin systems. Both *GSH* and *Trx1* has been linked to induction of pro-inflammatory macrophage phenotypes and increased phagocytic activity (42, 43). Upregulation of *Tgm2*, known to be induced in both pro- and anti-inflammatory differentiation and to be involved in phagocytosis (44), and of *Acss2*, a metabolic regulator recently implicated in histone acetylation (45), crotonylation (46) or lactylation (47), supports broad transcriptional and metabolic reprogramming in KO M0 macrophages. In M2 cells, upregulation of transcripts encoding for chloride (*Clcn5*) and sodium (*Slc9a9*) ion exchangers, which could impact phagocytic activity (48) as well as Zmat3, involved in RNA processing (49) indicate distinctive altered functions.

Further complementing the potentially direct effects of miR-210 targeting described above, context-dependent regulation led to the differential enrichment of pathways between M0 and M2 polarization states. In M0 macrophages, the positive control on cell cycle checkpoint pathways by up-regulated *Stmn1*, *Mt2*, and *H2ax*, involved in cycle arrest (50–52), complemented the reduced proliferation findings of our assays. Positive regulation of MYC and mTORC1 indicates metabolic reprogramming, which may be linked to regulation of inflammatory cytokine production (53) with *Egln3 (PHD3*) and *Slc2a1 (GLUT1*) potentially driving this pro-inflammatory state in miR-210 KO M0 cells (54, 55). Interestingly, numerous elements in response to ROS including Catalase, Gclm, a glutamate cysteine ligase subunit necessary for GSH synthesis, and reductases *Gsr*, *Srxn1*, *Prdx6* and *Txnrd1*, are also positively regulated. Redox signaling and ROS production are known to play important roles in macrophage function and in polarization towards both pro-inflammatory and reparative states. Yet the precise mechanisms directing their effects in polarization remain unclear (42). The elevated levels of antioxidant genes suggest an increase in oxidative stress. This could potentially drive or stem from the pro-inflammatory phenotype observed in M0 miR-210 KO macrophages, i.e., increased secretion of IL-6 and TNFα and IL-1β, compared to WT cells. These findings suggest that miR-210 plays a crucial role in suppressing excessive inflammatory responses, maintaining macrophage immune homeostasis.

The anti-inflammatory role of miR-210 has also been documented by other groups in various experimental settings. For example, miR-210 overexpression in RAW264.7 murine macrophages reduced TNFα and IL-6 levels upon *in vitro* stimulation with LPS (56). Similarly, lentiviral-induced miR-210 overexpression in a rat model of osteoarthritis led to decreased IL-1β, IL-6 and TNF-α levels in synovial fluid, while enhancing chondrocyte survival and suppressing NF-κB signaling via p65 downregulation (57), further supporting a protective role of miR-210 in chondrocytes during inflammatory conditions. Similar to the report by Zhang et al., we found a protective role of miR-210 in M2 macrophages, while the impact of miR-210 loss was negative regulation of multiple inflammatory pathways was less pronounced than in M0 macrophages. Specifically, miR-210 KO M2 macrophages exhibited suppressed stress response and inflammatory pathways, coupled with activation of the p53 pathway and increased apoptosis-related gene expression, further supported by elevated p53 protein levels. Additionally, these cells showed increased TGFβ protein levels, suggesting a senescence-like phenotype mediated by p53 activation, potentially leading to reduced proliferation and cell cycle arrest. Notably, TGFβ signaling has been previously demonstrated to enhance p53 stability, promoting its accumulation and subsequent regulation of apoptosis, cell cycle arrest, and DNA repair genes (58). However, the reduced proliferation in miR-210 KO cells is not a selective defect associated with M2 polarization, pinpointing miR-210 as a regulator of expansion rather than differentiation. This pattern mirrors our recently published data showing that miR-210 deletion reduced proliferation in non-immune cell types (59), reinforcing a broader, lineage-independent role for miR-210 in cell-cycle control.

This context dependence echoes our earlier work demonstrating a pro-fibrotic phenotype in miR-210-overexpressing macrophages *in vitro*, along with a protective role in ischemic muscle tissue, where miR-210 reduced inflammation, enhanced capillary density, and promoted tissue repair (60). However, in a hind limb ischemia model using chimeric mice transplanted with miR-210-overexpressing bone marrow cells, Zaccagnini et al. demonstrated that specific overexpression of miR-210 in hematopoietic cells led to dysregulated angiogenesis, increased inflammation, and impaired tissue repair, accompanied by fibrosis. These data converged towards a context-dependent regulation of the inflammatory response by miR-210.

Our study shows that miR-210 depletion yields divergent outcomes, driving an inflammatory gene signature in M0 macrophages, and a pro-fibrotic signature in M2-like cells, underscoring its integration into state-specific regulatory networks. Such duality is not unique to miR-210: similar context-specific outcomes have been well described for TGF-β, which exerts either pro-inflammatory or pro-fibrotic effects depending on the cellular and environmental context (61). Our data therefore support a model in which miR-210 functions as a molecular rheostat whose impact on macrophage fate is dictated by the prevailing metabolic, cytokine and transcriptomic configuration.

Interestingly, despite the transcriptional upregulation of glycolytic and oxidative metabolism components, a reduction in basal glycolytic activity observed specifically in M0 macrophages, along with a preserved oxygen consumption rate (OCR) and a comparable energetic profile under stress conditions. This contrast could indicate partial or transitional metabolic adaptation in M0 miR-210-deficient macrophages. Reduced glycolytic activity has also been observed in diabetic wounds and in primary human fibroblasts, where hyperglycemia suppresses miR-210 expression—a defect that can be reversed by restoring miR-210 levels (18). Similar glycolytic alterations have been reported in other cell types by Grosso et al, who showed that miR-210-expressing cancer cells exhibit a glycolytic phenotype (62). Notably, the observed dysregulation in hypoxia response pathway upon miR-210 loss is plausible, as Grosso et al. also demonstrated that miR-210 and HIF-1α exist in a positive feedback loop, where an increase of miR-210 can stabilize HIF-1α.> Kieran et al. demonstrated that miR-210 overexpression led to increased glycolytic activity in astrocytes, concomitant with reduced pro-inflammatory markers, such as complement 3 and Semaphorin 5b proteins, associated with neurotoxic astrocytes (63). Noteworthy, despite reduced glycolysis, miR-210 KO macrophages displayed significantly increased phagocytic activity in both M0 and M2 polarization states, suggesting a compensatory adaptation to maintain immune functionality under metabolic constraints (1, 64, 65).

Pathway enrichment analysis of genes differentially expressed during macrophage polarization suggested an incomplete M2 polarization in miR-210 KO cells under IL-4 stimulation. The absence of significant changes in genes involved in a couple of biological processes specific for cell growth and proliferation pathways aligns with the observed reduction in cycling cells and decreased proliferation rates. Similar regulatory effects were also reported with other cells, in which loss of miR-210 altered a variety of cellular phenotypes including proliferation and apoptosis (66), thus reinforcing its role in macrophage differentiation and functional specialization.

Collectively, these findings highlight the multifaceted role of miR-210 in macrophages, regulating inflammatory responses, metabolic function, cell cycle progression, and polarization dynamics. The observed alterations in miR-210 KO macrophages suggest that miR-210 is a key modulator of macrophage function, with potential implications for chronic inflammatory diseases, immune dysregulation, and tissue repair.

## Data Availability

The datasets presented in this study can be found in online repositories. The names of the repository/repositories and accession number(s) can be found below: https://www.ncbi.nlm.nih.gov/geo/, GSE296928.
