# Supplementary material for: miR-210 promotes the anti-inflammatory phenotype and M2 polarization in murine macrophages
Source: Front Immunol. 2025 Aug 5;16:1633163. doi: 10.3389/fimmu.2025.1633163 (PMC12361208; doi:10.3389/fimmu.2025.1633163)
Supplement: Supplementary file 1 [file DataSheet1.docx]

Supplementary Material

*
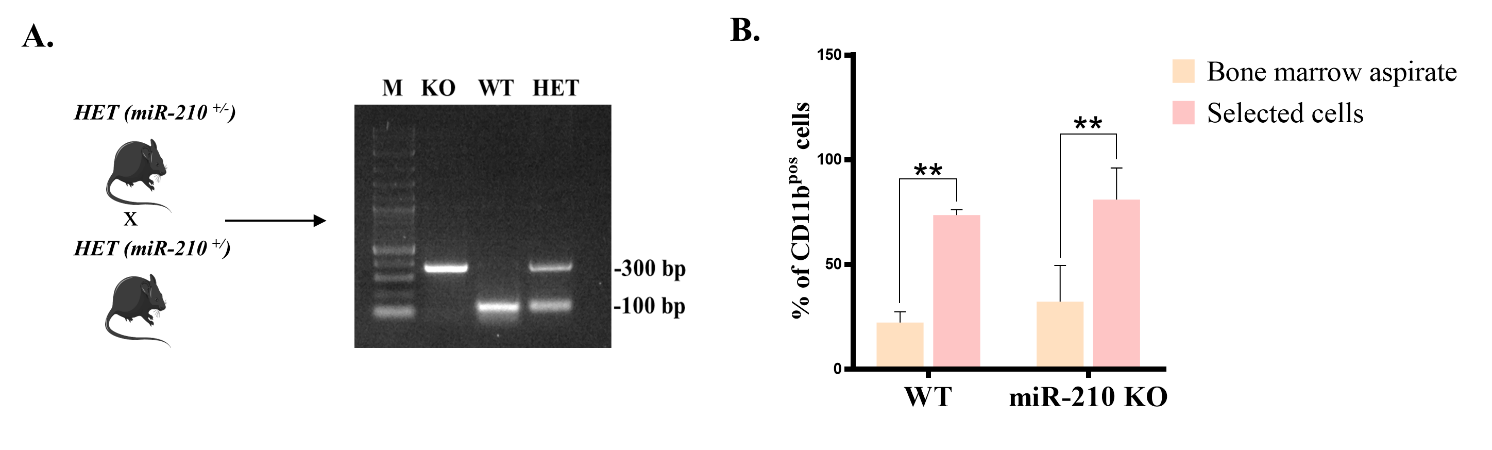
*

**Supplementary Figure 1.** (A)Validation of miR-210 deletion in macrophages. (B) Quantification of CD11b⁺ cells from bone marrow aspirates and selected cell populations in WT and miR-210 KO mice. Data are expressed as percentage of CD11b⁺ cells within total bone marrow-derived cells before and after magnetic selection. Results represent three independent experiments. Statistical analysis was performed using two-way ANOVA, comparing bone marrow aspirates vs selected cells within each genotype. p < 0.01 (**).

**
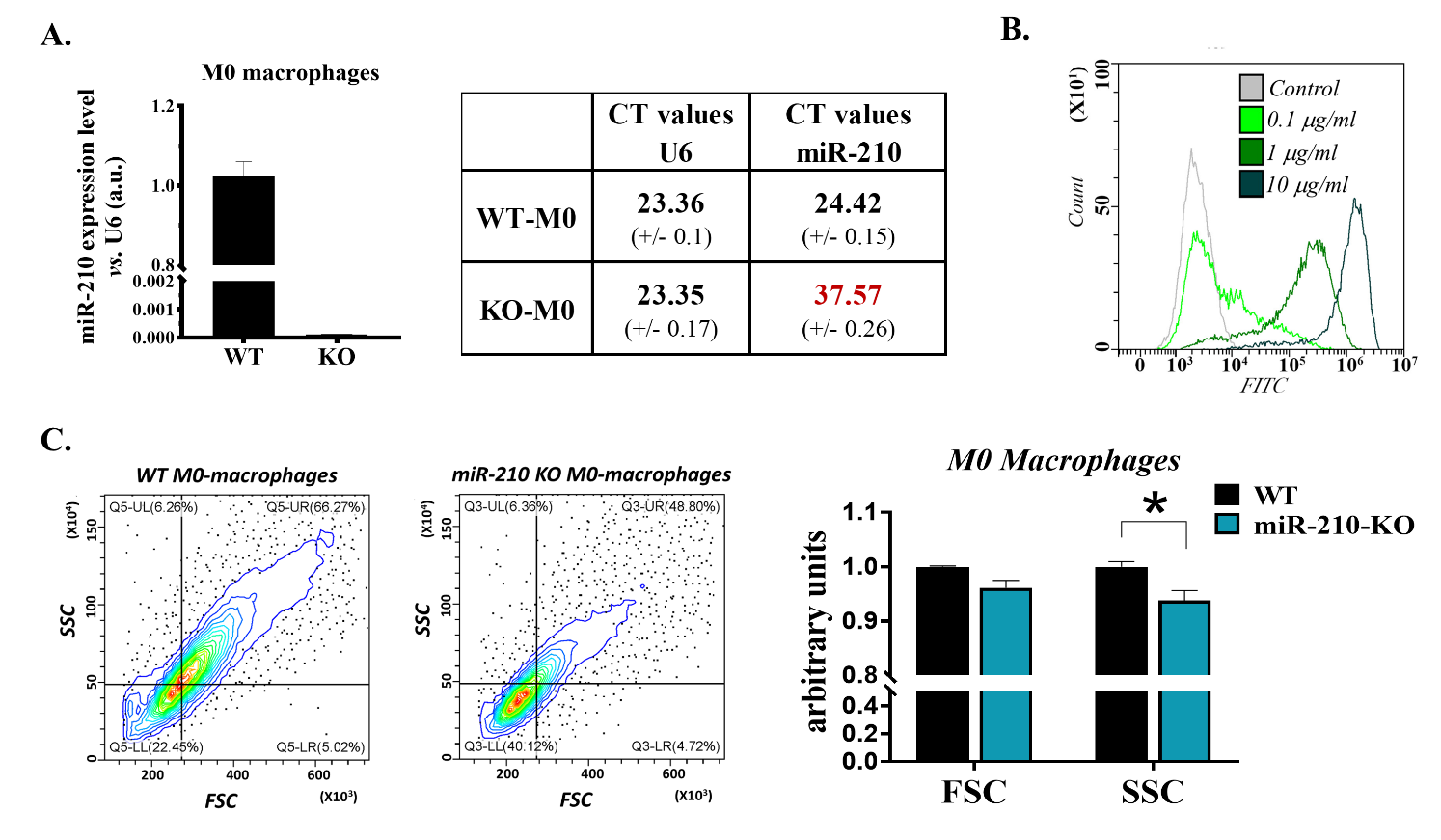
**

**Supplementary Figure 2. (A)** Validation of miR-210 -KO in macrophages by real-time qPCR analysis using TaqMan assay probes. Expression levels of miR-210 were normalized to U6 and showed a drastic reduction in KO cells compared to WT. The table on the right presents CT values for U6 and miR-210, confirming the successful deletion of miR-210 in KO macrophages. (B) Flow cytometry analysis illustrating the phagocytic uptake of S. aureus bioparticles by M0 macrophages. (C) Scatter plots illustrating the size (FSC) and granularity (SSC) of WT and miR-210 KO macrophages. The histogram on right shows the comparative analysis, revealing a significant difference in SSC values between miR-210-KO and WT cells. Data are presented as the means ± SEMs of 3 independent experiments. Statistical significance was determined using two-way ANOVA, *p < 0.05.

**
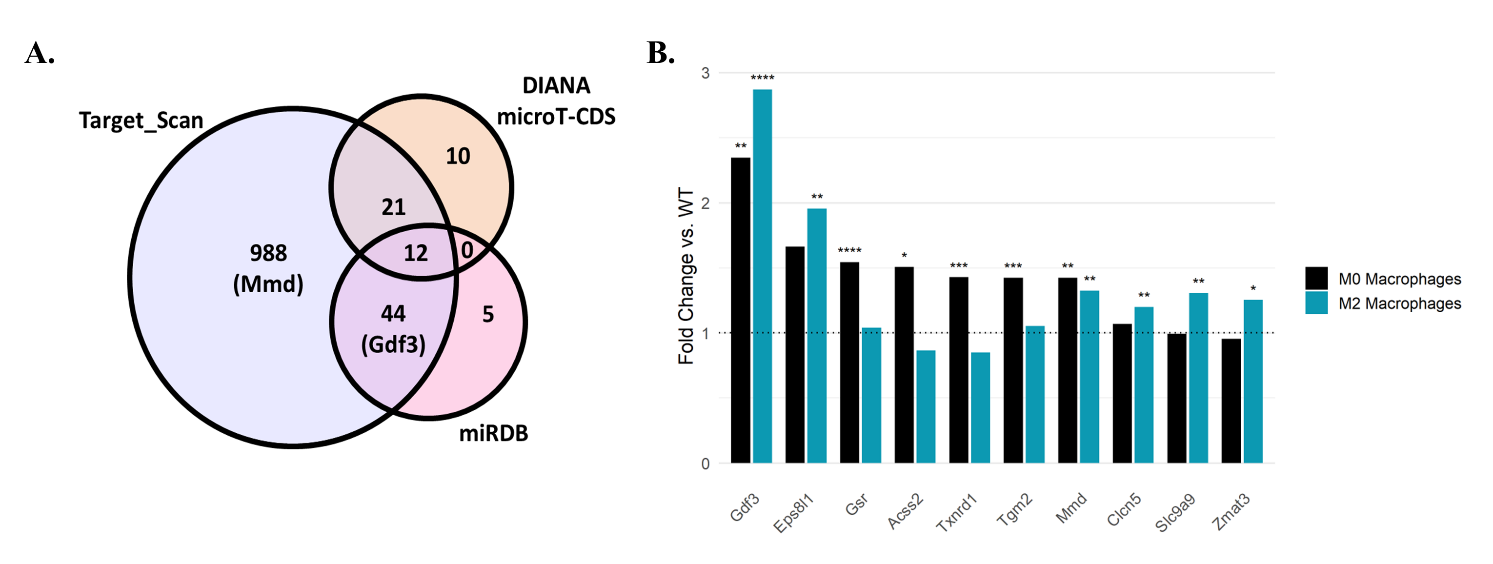
**

**Supplementary Figure 3.** Identification of predicted miRNA targets within DEGs. (A) Venn Diagram illustrating the miR-210 targets predicted by three sources: TargetScan, DIANA microT-CDS, and miRDB. (B) Bar graph of predicted miR-210 targets that were identified as significantly differentially up-regulated upon miR-210 loss in M0 and/or M2 macrophages. Data is presented as fold change of KO vs. WT state. Statistical significance (DESeq2 analysis) is indicated as follows: **** p < 0.0001, *** p < 0.001, ** p < 0.01.

**
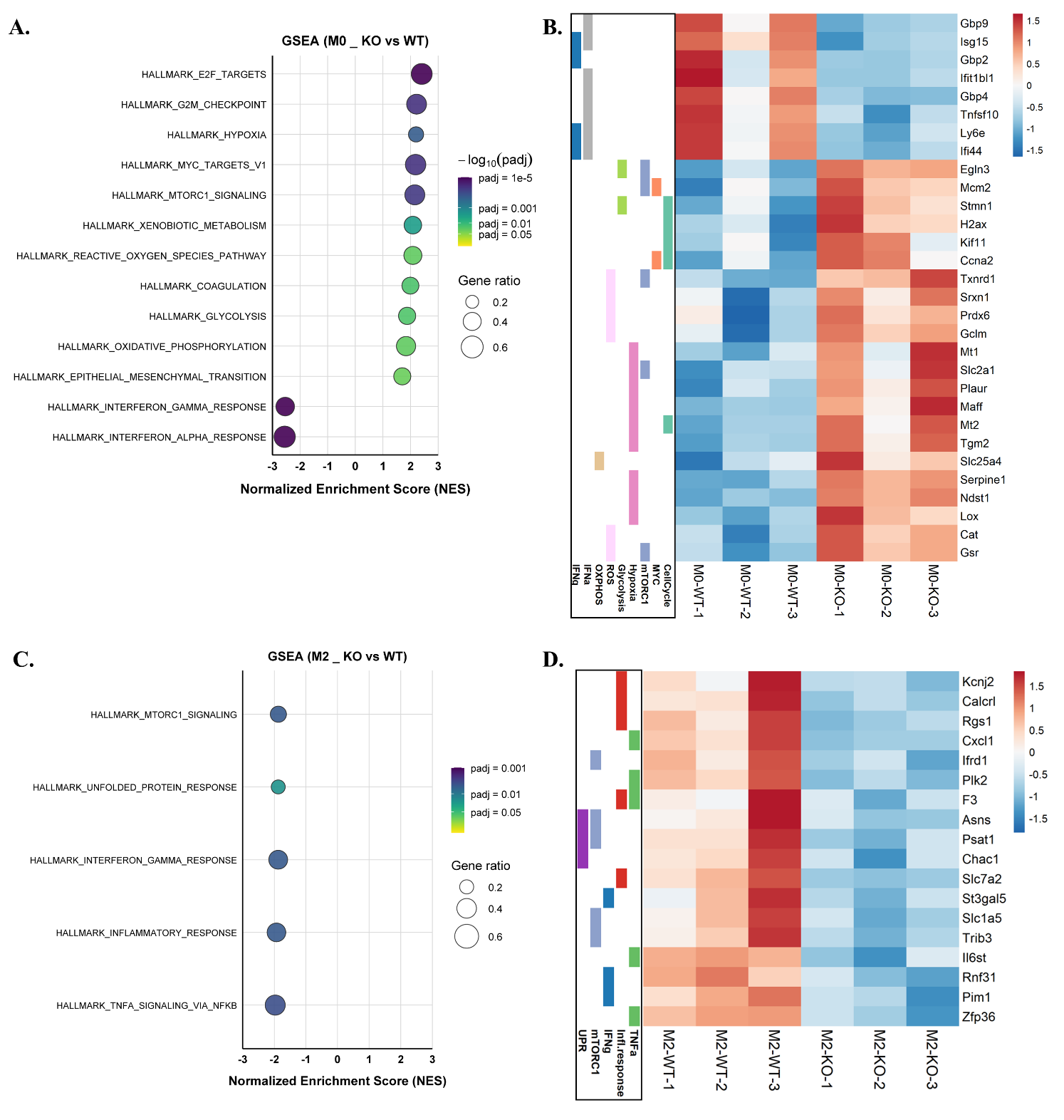
**

**Supplementary Figure 4.** Gene set enrichment analysis (GSEA) results for miR-210 KO *vs.* WT macrophages. (A) Significant (FDR<0.05) positive and negative pathways in M0 macrophages. (B) Heatmap of DEGs that contributed to the positive/negative Normalized Enrichment Score (NES) for pathways appearing as significant (FDR<0.05) in M0 cells: E2F Targets, G2M Checkpoint, MYC Targets, mTORC1 Signaling, Hypoxia, Glycolysis, Reactive Oxygen Species, Oxidative Phosphorylation, IFN-a and IFN-g Response. (C) Significant (FDR<0.05) positive and negative pathways in M2 macrophages, ordered by significance. (D) Heatmap of DEGs that contributed to the negative NES for pathways appearing as significant (FDR<0.05) in M2 cells: TNF-a Signaling via NF-kB, IFN-g Response, mTORC1 Signaling, Inflammatory Response, Unfolded Protein Response (UPR). MSigDB pathways with a positive NES feature genes with expression levels skewed towards up-regulation upon miRNA loss, and vice versa. Genes included in heatmaps are annotated with regards to their involvement in each of the significant pathways.

**
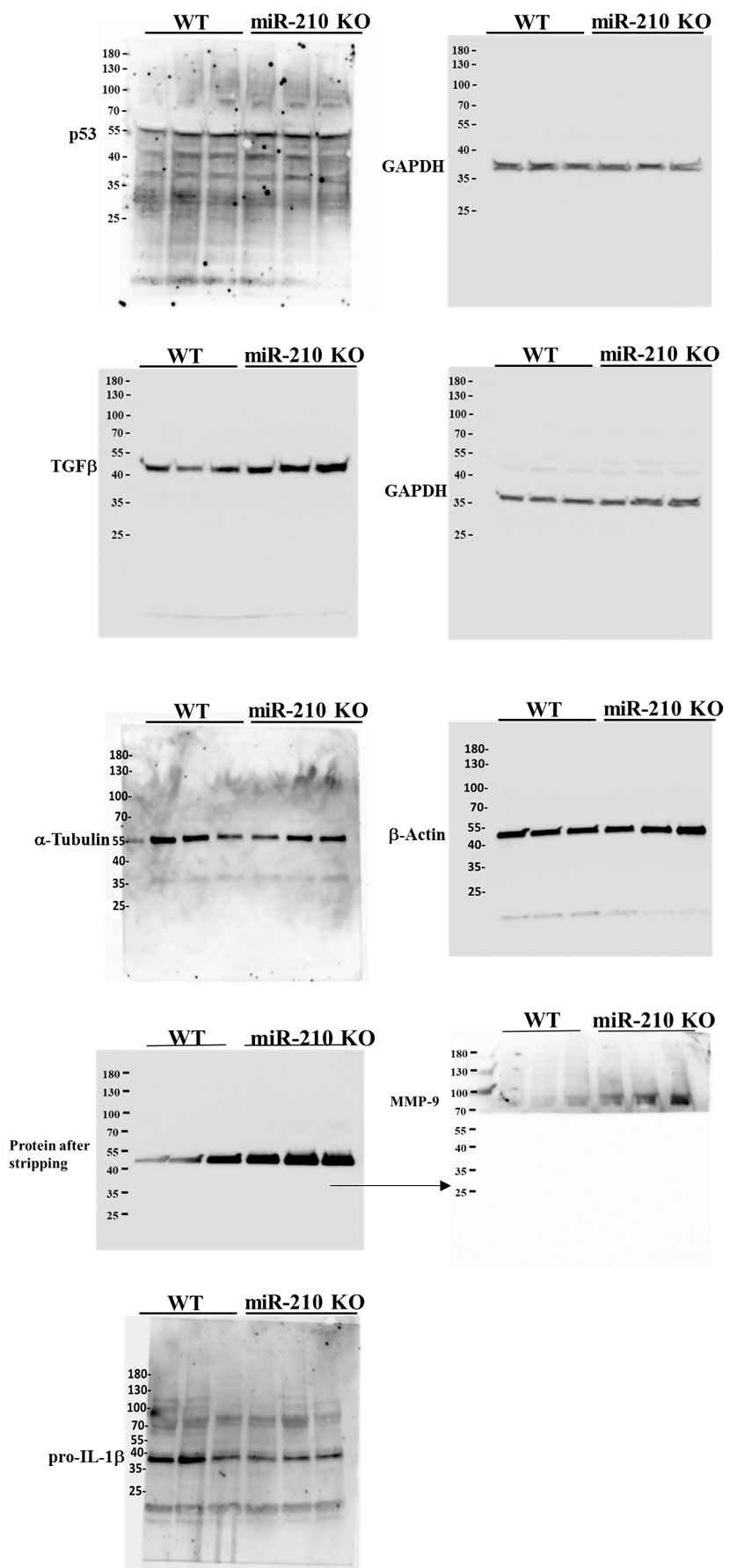
**

**Supplementary Figure 5.** Uncropped Western blot images related to Figures 4 and 5.
